# The Effect of a Fortified Date Bar on Perceived Stress and Morning Salivary Cortisol in Female Students: A Pilot Randomized Controlled Trial

**DOI:** 10.3390/nu18193134

**Published:** 2026-09-23

**Authors:** Evgeniya Klein, Daria Velina, Sherzodkhon Mutallibzoda, Svetlana Tefikova, Olga Orlovtseva, Natalia Lebedeva, Igor Nikitin

**Affiliations:** 1 Department of Food Technology and Bioengineering, Plekhanov Russian University of Economics, 36 Stremyanny per., 115054 Moscow, Russia; kattim67@gmail.com (D.V.); mutallibzoda.s@rea.ru (S.M.); tefikova.sn@rea.ru (S.T.); orlovtseva.oa@rea.ru (O.O.); 2 Department of Innovative Technologies of Plant-Based Products, K.G. Razumovsky Moscow State University of Technologies and Management (The First Cossack University), Zemlyanoy Val, 73, 109004 Moscow, Russia; n.lebedeva@mgutm.ru; 3 Department of Biotechnology of Food Products from Plant and Animal Raw Materials, K.G. Razumovsky Moscow State University of Technologies and Management (The First Cossack University), Zemlyanoy Val, 73, 109004 Moscow, Russia

**Keywords:** perceived stress, salivary cortisol, magnesium, vitamin C, date bar, fortified food, university students, randomized controlled trial, nutritional intervention, HPA axis

## Abstract

**Background/Objectives**: Chronic stress poses a serious threat to public health and is associated with the rising global burden of mental disorders, including anxiety and depression. University students, particularly young women, represent a highly vulnerable group due to the combination of academic pressure, social adaptation challenges, and gender-specific stress reactivity. This pilot randomized controlled parallel-group trial aimed to evaluate the effect of a 4-week daily consumption of a date bar fortified with magnesium (240 mg), vitamin C (300 mg), inulin (1000 mg), and grape seed extract (100 mg) on perceived stress and morning salivary cortisol in healthy female university students under natural academic load. **Methods**: Thirty healthy female students (aged 18–28 years) were randomized in a 1:1 ratio to either the intervention group (fortified date bar) or the control group (placebo bar) for 4 weeks. The primary outcome was the change in morning salivary cortisol levels (measured 30–60 min after awakening), and the secondary outcome was the change in perceived stress assessed by the Perceived Stress Scale (PSS-14). Measurements were performed at baseline and within 24–48 h after the intervention. Adherence was monitored via daily digital reports and weekly in-person meetings. Statistical analysis was performed using non-parametric tests (Mann–Whitney U test, Wilcoxon test) with effect sizes (rank-biserial correlation, r). Per-protocol analysis was applied for the primary outcome (*n* = 28) and available-case analysis for the secondary outcome (*n* = 29). Participants and outcome assessors were blinded to group allocation throughout the study. **Results**: No adverse events were reported. Median adherence was 100% in both groups, with all participants demonstrating ≥ 90% compliance. No statistically significant changes in morning salivary cortisol were observed within or between groups (within-group: *p* = 0.173 in the intervention group, *p* = 0.326 in the control group; between-group: *p* = 0.104), although a consistent trend toward a reduction was observed in the intervention group (median Δ = −0.64 ng/mL) with a moderate effect size (*r* = 0.43 within-group; *r* = −0.37 between-group). For perceived stress, a statistically significant reduction in PSS-14 scores was observed within the intervention group (median Δ = −4.0 points; *p* = 0.009) with a large effect size (*r* = 0.80), while no significant change occurred in the control group (*p* = 0.624). Between-group comparison for PSS-14 changes did not reach statistical significance (*p* = 0.073), although the effect size was moderate (*r* = −0.40). No significant correlation was found between changes in cortisol and changes in PSS-14 scores (ρ = −0.134; *p* = 0.496). **Conclusions**: In this pilot randomized controlled trial, four-week consumption of a fortified date bar was associated with a reduction in perceived stress among healthy female university students, as reflected by a large within-group effect size on PSS-14 scores. However, given the exploratory nature of the study, the small sample size, and the absence of an a priori power calculation, this finding should be regarded as preliminary and hypothesis-generating rather than as evidence of established efficacy. A consistent, albeit non-significant, trend toward reduced morning cortisol was also observed, suggesting a possible modulatory effect on the HPA axis that requires confirmation in larger, longer-term studies with optimized dosages. The dissociation between subjective and objective stress measures underscores the multidimensional nature of the stress response and supports the use of complementary assessment approaches in future research. The fortified date bar may warrant further investigation as a potentially feasible, acceptable, and economically accessible component of primary stress prevention strategies in young populations, although this possibility remains to be tested in adequately powered confirmatory trials.

## 1. Introduction

The problem of chronic stress has acquired the character of a non-communicable pandemic of the 21st century, affecting developed and developing countries alike [1]. The increasingly competitive interpersonal and professional relationships, geopolitical and economic instability, as well as the exponential growth of cognitive load mediated by the digitalization of everyday life, collectively generate a persistently negative psychological and emotional state [2,3,4]. Adolescents and young people demonstrate particular vulnerability to the aforementioned factors, which is associated with the incomplete ontogenetic maturation of the prefrontal cortex and heightened sensitivity to socially evaluative stimuli [5,6].

Systemic activation of the hypothalamic–pituitary–adrenal (HPA) axis induces a sustained elevation of basal cortisol levels, which is associated with a broad spectrum of pathophysiological responses—ranging from impaired neuroplasticity in the hippocampus [7] to excessive release of pro-inflammatory cytokines by immune cells [8]. Thus, stress contributes substantially to the global burden of mental disorders. At the same time, despite the high prevalence of clinically significant anxiety and depressive symptomatology, the rate of help-seeking for specialized psychotherapeutic and psychiatric care remains critically low, particularly in developing countries [9,10]. This is attributable, firstly, to the persistent social stigmatization of mental disorders, which is especially pronounced in conservative cultures [11]; secondly, to the objective insufficiency of government-funded screening and early intervention programs [12,13]; and thirdly, to the high cost of qualified long-term psychotherapy, which requires a personalized approach and substantial resource expenditure not always covered by compulsory health insurance systems [14,15].

The aforementioned limitations necessitate the search for alternative, economically accessible, and physiologically grounded strategies for the primary prevention of chronic stress—strategies that can be integrated into daily life without requiring significant behavioral restructuring on the part of individuals. A promising avenue for such prevention lies in the development of specialized food products enriched with bioactive compounds that possess proven anti-stress potential (nutrient adaptogens and mood stabilizers).

Among the micronutrients with potential anti-stress activity, L-ascorbic acid (vitamin C) is considered, acting as a key cofactor for enzymes involved in catecholamine biosynthesis [16] and a modulator of the adrenergic response [17]. Vitamin C may prevent excessive glucocorticoid production in the body during stress [18]; moreover, according to research, ascorbic acid supplements contribute to reducing fatigue and increasing work engagement [19], exert anxiolytic and stress-suppressing effects [20], improve mood in subclinical forms of depression [21], and facilitate faster normalisation of cortisol levels following acute psychological stress exposure [22]. The potent antioxidant properties of vitamin C underpin its role in protecting neurons from oxidative damage [23], while its positive influence on gut microbiota helps reduce inflammation and supports mental health via the gut–brain axis [24].

Magnesium is regarded as an endogenous physiological antagonist of calcium channels and a modulator of the NMDA receptor system, which underlies its neuroprotective and sedative properties and explains the close association between magnesium deficiency and such pathological conditions as migraine, Alzheimer’s disease, Parkinson’s disease, depression, and anxiety [25]. Among the putative mechanisms linking magnesium to mental disorders, dysregulation of the HPA axis is also cited, manifesting as elevated adrenocorticotropic hormone levels under conditions of magnesium deficiency [26]. Magnesium supplements have repeatedly demonstrated efficacy in achieving clinical improvements in patients with depression and anxiety disorders [27], while high dietary magnesium intake correlates with a reduced risk of depressive disorders at the population level [28]. Equally important is the role of magnesium in maintaining sleep quality: mineral supplementation positively affects sleep efficiency and duration in adult populations [29], thereby counteracting the accumulation of fatigue and enhancing stress resilience. Interestingly, recent studies have further linked magnesium’s contribution to mental health maintenance with modulation of the gut–brain axis: according to data [30], magnesium consumption increases microbiome diversity and enhances the synthesis of short-chain fatty acids—metabolites of gut bacteria that possess neuroactive properties [31].

Taken together, this evidence suggests that fortification of food products with vitamin C and magnesium may potentially target the stress response system through mechanisms of neuroendocrine regulation at the level of the HPA axis and the central nervous system, through the enhancement of anti-inflammatory and antioxidant activity, as well as through modulation of the gut–brain axis. Additional nutritional components in this context may include sources of dietary fiber and polyphenolic compounds, which would augment the prebiotic and anti-inflammatory potential of the food matrix [32,33]. The incorporation of dietary fiber into the fortified product appears particularly important in light of the substantial body of evidence associating fiber consumption with a reduced risk of depression and anxiety disorders [34,35], as well as in view of the concerning data on the widespread deficiency of dietary fiber in the diets of populations in both developed and developing countries [36,37].

In the present study, inulin—a polysaccharide derived from chicory roots (*Cichorium intybus*)—was selected as the source of dietary fiber. This prebiotic has previously demonstrated promising anti-inflammatory effects: in animal models, chicory consumption was shown to reduce the expression of inflammatory cytokines in the intestine [38], particularly under stress conditions [39], which may potentially attenuate the negative impact of psycho-emotional tension on the central nervous system via the gut–brain axis. An equally important criterion for selecting inulin is its technological compatibility with the fruit bar matrix [40], its ability to retain prebiotic properties within various food products [41], and its improved glycemic index [42].

A standardized grape seed extract (with a polyphenol content of at least 95%) was selected as the source of polyphenolic compounds. When used outside a food matrix (in tablet form), this ingredient has previously demonstrated the ability to improve mood and reduce perceived stress [43], to enhance the overall antioxidant function of the body [44], and, in cell model studies, to exhibit anti-inflammatory properties through a simultaneous decrease in the expression of inflammatory cytokines and an increase in the expression of anti-inflammatory cytokines [45].

The target consumer group for the present study comprises female university students aged 18–28 years, as this cohort is characterized by the simultaneous presence of multiple stress-related risk factors: firstly, as noted above, young adults are more vulnerable to stress, anxiety, and depression compared with older age groups [5,6,46]; secondly, gender studies have demonstrated that women in early adulthood exhibit higher levels of chronic stress and tension than their male counterparts [47]; and thirdly, university education creates an environment of heightened psycho-emotional burden, driven both by changes to one’s habitual way of life (relocation from the parental home to dormitories or rented accommodation) and by academic pressure [48,49].

A date-paste-based bar has been selected as the carrier product, which aligns with the preferences of the target group across the following parameters: convenience and speed of consumption, small size and portability for academic settings, and the absence of added sugar and preservatives. Owing to the sustained trend towards healthy eating, date bars have become a familiar product among the population, and their consumption continues to grow [50], making their large-scale fortification a promising strategy for nutritional prevention.

The primary objective of the present study is to evaluate the effect of regular consumption of date bars fortified with magnesium citrate, L-ascorbic acid, inulin, and grape seed extract over a period of 4 weeks on the level of subjectively perceived stress and on salivary cortisol concentration in the morning portion of saliva among female university students under conditions of natural academic load. Salivary cortisol has been preselected as an objective biochemical marker of stress, taking into account a number of fundamental and methodological advantages. Firstly, in contrast to blood serum, saliva demonstrates high validity in capturing stress reactivity with minimal procedural invasiveness [51]; secondly, saliva can be collected at home, thereby enabling strict temporal alignment with the circadian rhythm of cortisol (peak secretion occurring within 30–45 min after awakening [52]); thirdly, salivary cortisol is characterized by high stability at room temperature and under refrigerated storage [53], which simplifies the logistics of sample collection and transport within the context of a pilot study.

The dosage of the bar’s active components was determined taking into account their potential influence on the sensory characteristics of the product and the technological feasibility of incorporating a given proportion of each ingredient into the food matrix. Accordingly, the selected doses of the active components are somewhat lower than those used in most monotherapy studies of the corresponding nutrients:

- vitamin C—300 mg per serving: in most studies of the potential effect of ascorbic acid on mental well-being, a dosage of 500–1000 mg/day has been adopted [54,55];
- magnesium citrate—1.5 g per serving (240 mg of elemental magnesium): in studies of the effect of magnesium on anxiety and subjective stress levels, the dosage range varied from 46.4 to 600 mg/day [56]; in a study of the effect of magnesium on glucocorticoid metabolism, a dosage of 350 mg/day was used [57];
- inulin—1 g per serving: in a number of studies examining the effect of inulin on inflammation and depressive symptoms, a dose of 9.2–10.0 g/day is reported [58,59,60];
- grape seed extract—100 mg per serving: in studies of the effect of grape polyphenols on oxidative status, mood, and stress indicators, dosages of 250–300 mg/day are used [43,61,62].

Within a single food matrix, an additive effect is expected from the combined use of active substances acting on different links of the stress response: magnesium and vitamin C act jointly on neuroendocrine regulation and the HPA axis, while inulin and grape seed extract act on the microbiome, the epithelial barrier, and the oxidative and inflammatory components of stress. With such a combined exposure, the dose of each individual component was selected to be lower than that required for monotherapy, since the expected final effect is formed through complementary mechanisms rather than through a multiple increase in the concentration of a single nutrient.

To the best of our knowledge, no RCT has been conducted to date in which all four selected components were administered simultaneously within a single food product, with an assessment of their combined effect on subjective and objective markers of stress. At the same time, the combined format is of interest from two perspectives: first, as noted above, the components act on different but interrelated links of the stress response, providing an additive effect; second, the use of a date bar as the carrier makes it possible to integrate fortification into the habitual diet without the need to take several separate supplements, which is particularly relevant for a young female population with a high level of everyday stress and low adherence to long-term pharmacological interventions.

## 2. Materials and Methods

### 2.1. Trial Design

#### 2.1.1. General Information

The present study was conducted in accordance with a double-blind, randomized controlled trial (RCT) design with parallel groups and a 1:1 participant allocation ratio. The study was pilot in nature and was aimed at a preliminary evaluation of the effect of 4-week daily consumption of a date bar fortified with magnesium, vitamin C, inulin, and grape seed extract on morning salivary cortisol levels and perceived stress levels as measured by the PSS-14 scale in healthy young women.

There was no direct involvement of patients or the public in the design, conduct, or reporting of this trial.

The study protocol was registered with ClinicalTrials.gov, identification number NCT07585422 (https://clinicaltrials.gov/study/NCT07585422, accessed on 3 May 2026). The study protocol was registered on 7 May 2026, prior to the enrollment of the first participant. The first participant was enrolled on 22 May 2026.

#### 2.1.2. Study Conditions and Ethical Considerations

The organization of participant recruitment, the conduct of the intervention, and the collection of primary data were carried out by members of the research team at the Department of Food Technology and Bioengineering of Plekhanov Russian University of Economics (Head of Department—I.N.) during the period from May 2026 to July 2026. Ethical review of the study protocol was conducted by the Local Ethics Committee (LEC) of the Federal State Budgetary Educational Institution of Higher Education “K.G. Razumovsky Moscow State University of Technologies and Management (First Cossack University)”. This committee was selected because, at the time of study planning, no active ethics committee was available at Plekhanov Russian University of Economics. The protocol was approved at a meeting of the LEC of MSUTM (Protocol No. 6 dated 5 May 2026). The dual affiliation of the principal investigator (I.N.) with both institutions is disclosed for transparency; the ethics committee reviewed the protocol independently, in accordance with its standard procedures and the Declaration of Helsinki, and the principal investigator did not participate in the committee’s deliberation or decision-making process.

All participants were thoroughly informed of the aims and procedures of the study, potential risks, and the right to voluntarily withdraw from participation at any stage. Written informed consent was obtained from each participant prior to the initiation of any study-related procedures. The study was conducted in full compliance with the ethical principles of the Declaration of Helsinki of the World Medical Association (1964, with amendments up to 2013).

#### 2.1.3. Randomization and Group Formation

To ensure comparability of groups with respect to baseline salivary cortisol levels, stratified randomization was applied. The procedure was conducted in several sequential stages.

In the first stage, all participants who passed the initial screening according to inclusion/exclusion criteria (*n* = 34) provided morning saliva samples for determination of basal cortisol levels (collection was carried out 30–60 min after waking up between 6:00 and 10:00). After receiving the laboratory analysis results, 30 participants who met all inclusion/exclusion criteria were ranked according to the value of the baseline cortisol level (ng/mL). Based on the sample median value, participants were divided into two strata:

Stratum A—participants with cortisol levels at or above the median value (*n* = 15);

Stratum B—participants with cortisol levels below the median value (*n* = 15).

At the second stage, participants within each stratum were allocated to either the intervention or control group using simple randomization with a random number generator. Randomization was performed by an independent researcher (S.T.), who was not involved in participant recruitment or subsequent outcome assessment. The RAND function of Microsoft Excel (Version 2412, Build 18324.20190) was used to generate the random sequence. The procedure was carried out as follows:

1. In a Microsoft Excel spreadsheet, each participant within the strata was assigned a sequential number from 1 to 15.
2. For each participant, a random number between 0 and 1 was generated using the function = RAND(), with precision to 10 decimal places.
3. Participants within each stratum were ranked in ascending order of the generated random number (sorting by the column containing the random numbers).
4. The first 8 participants in the sorted list in Stratum A were assigned to the intervention group, and the next 7 to the control group. In Stratum B, the allocation was performed in a mirrored fashion: the first 7 participants were assigned to the intervention group, and the next 8 to the control group.

As a result of the randomization procedure, two groups of 15 participants each were formed:

- Intervention group—participants receiving a date bar fortified with magnesium (240 mg of elemental magnesium in the form of citrate), vitamin C (300 mg in the form of L-ascorbic acid), inulin (1000 mg), and grape seed extract (100 mg). This group comprised 8 participants from Stratum A and 7 participants from Stratum B.
- Control group (placebo group)—participants receiving a placebo bar, matched as closely as possible in organoleptic properties but devoid of the aforementioned active components (replaced with maltodextrin). This group comprised 7 participants from Stratum A and 8 participants from Stratum B.

#### 2.1.4. Blinding

To ensure allocation concealment, a system of opaque sealed envelopes was employed. Upon completion of the randomization procedure, an envelope was prepared for each participant, with the outer side bearing an individual identification number (a five-digit code). Inside the envelope was an indication of group assignment (“Intervention” or “Control”). The envelopes were sealed and handed over to the researcher responsible for bar distribution (D.V.). This researcher was not involved in randomization, participant recruitment, or outcome assessment. Each participant received a bar of the corresponding type only after her individual envelope had been opened immediately on the day of intervention commencement. The allocation sequence was concealed from all members of the research team until the actual distribution of bars, with the exception of the researcher who performed randomization (S.T.), who had no further interaction with participants at subsequent stages.

Participants were informed that the study was placebo-controlled, but their assignment to either the intervention or control group was concealed from them. Research personnel directly interacting with participants (bar distribution, counselling, questionnaire collection) were likewise unaware of group allocation. Assessment of the objective biomarker (salivary cortisol levels) was conducted at an independent accredited medical laboratory, LLC “CHROMOLAB” (Moscow, Russia). Laboratory staff performing HPLC-MS/MS were not informed of sample group allocation, thereby ensuring blinding at the laboratory analysis stage. Thus, the study was conducted according to a double-blind design, with blinding of both participants and personnel.

#### 2.1.5. Protocol Amendments After Study Initiation

No substantial modifications to the study design were introduced after commencement. All procedures, inclusion and exclusion criteria, and methods of data collection and analysis were in accordance with the approved protocol.

Of the 34 participants who underwent initial screening, 4 were excluded prior to the randomization stage based on biochemical analysis results: morning salivary cortisol levels were below the threshold value of 1.5 ng/mL, which constituted an exclusion criterion stipulated by the protocol. These participants were advised to seek medical consultation from a relevant specialist. Accordingly, 30 participants (15 in each group) proceeded to the 4-week intervention.

During the course of the study, 2 participants withdrew: one from the control group due to the initiation of pharmacological therapy affecting cortisol levels, which constituted a post hoc exclusion criterion; and one from the intervention group due to inability to provide a final saliva sample in a timely manner because of an acute respiratory illness. Analysis of the primary outcome (cortisol levels) included only those participants who completed the full protocol and provided all planned measurements at both time points (*n* = 28, 14 in each group). For the secondary outcome (PSS-14), an available-case analysis was applied, including the participant from the intervention group who provided final questionnaire data (*n* = 29, 15 in the intervention group and 14 in the control group). All other design parameters (intervention duration, biomaterial collection methods, psychometric assessment tools, statistical analysis) remained unchanged.

### 2.2. Participants

Participant recruitment was conducted in May 2026 at the Department of Food Technology and Bioengineering of Plekhanov Russian University of Economics. Information about the study was disseminated through social media and messaging platforms. Healthy women aged 18–28 years, residing in Moscow or the Moscow Region, with a body mass index (BMI) in the range of 17.0–29.9 kg/m^2^, who had no diagnosed mental disorders or chronic endocrine diseases, and who were not taking glucocorticoids, hormonal preparations, or dietary supplements containing magnesium, vitamin C, or phytochemical compounds at the time of screening, were invited to participate. A complete list of inclusion and exclusion criteria is provided in Table 1.

The exclusion criterion for participants with cortisol levels below 1.5 ng/mL warrants particular attention. From a physiological standpoint, morning salivary cortisol concentrations below this level may indicate suppression of HPA axis activity, which can result from a range of factors including chronic fatigue, subclinical adrenal insufficiency, or prolonged exposure to stressors leading to blunted cortisol reactivity [63]. Including such participants could confound the assessment of the intervention effect, since the expected direction of change in response to nutritional support may differ fundamentally between individuals with normal/high and suppressed HPA axis activity.

Screening was conducted in two stages. At the first (remote) stage, candidates completed an electronic questionnaire (Yandex Forms, Moscow, Russia) containing questions regarding age, place of residence, anthropometric data, presence of chronic diseases, current use of medications and dietary supplements, as well as the presence of food allergies. At the second (in-person) stage, candidates meeting the preliminary criteria were invited to the university, where they were provided with full oral and written information about the aims, procedures, potential risks, and the right to withdraw from participation.

### 2.3. Interventions

For the purposes of the study, two types of date bars weighing 25 g each were developed: a fortified bar for the intervention group and a placebo bar for the control group. Both products were manufactured at the R&D Centre for Food Biotechnology of Plekhanov Russian University of Economics by center staff and members of the research team (E.K., S.M.). The composition of the products is presented in Table 2.

The production of bars was carried out under laboratory conditions in compliance with sanitary and epidemiological requirements. The components used for bar manufacture had certificates of conformity and declarations of conformity confirming their safety for food use. Active ingredients (magnesium citrate, L-ascorbic acid, grape seed extract, inulin) were purchased from certified suppliers of food ingredients (LLC “Miopharm”, Moscow, Russia; LLC “Stoing”, St. Petersburg, Russia).

The nutritional composition of the bars was calculated based on the ingredient suppliers’ specifications and publicly available reference data; the dietary fiber content of inulin was 88%. Energy values were calculated using Atwater factors (protein 4 kcal/g, fat 9 kcal/g, available carbohydrates 4 kcal/g, polyols 2.6 kcal/g). Dietary fiber was not included in the energy calculation. Values are presented per 25 g bar (Table 3). Laboratory verification of the nutritional composition of the final product was not performed; therefore, the values should be considered approximate.

For the most part, differences in the nutritional composition of the two bars were minimal, with the exception of the target bioactive nutrients. However, to equalize weight and volume, maltodextrin was used in the placebo bar in place of the active components, which resulted in a somewhat higher content of total carbohydrates (17.1 g vs. 14.4 g) and, accordingly, energy (79 kcal vs. 70 kcal per bar). These differences were considered acceptable for the purposes of the present pilot study, since the increase in energy content corresponded to less than 0.5% of the reference daily intake (2000 kcal), and the increase in carbohydrate content to approximately 1% of the reference daily intake, which is not physiologically meaningful and does not introduce systematic bias between the groups.

Bar distribution was conducted weekly during in-person meetings at Plekhanov Russian University of Economics. At each distribution, participants were provided with a one-week supply of bars (7 pieces) in a black metallized zip-lock bag bearing a label with the participant’s individual identification number. Participants were instructed to consume one bar daily at any time of day, separately from foods containing phytates, tannins, and calcium (no earlier than 1.5–2 h after their consumption) to ensure optimal magnesium absorption. Participants were advised not to alter their habitual diet and physical activity, and to limit alcohol consumption throughout the intervention period due to its potential impact on HPA-axis activity and sleep quality.

To monitor adherence, all participants were included in closed chat groups on the messaging platforms Telegram and VK.com. Daily between 20:00 and 21:00, a member of the research team (E.K.), who was unaware of participants’ group allocation, sent a standardized reminder to consume the bar along with an electronic survey to confirm intake. In the event of a missed dose, participants reported this to the research team member (E.K.).

Adherence to the intervention was calculated individually for each participant as the ratio of the number of bars consumed (confirmed by electronic survey and returned wrappers) to the number dispensed (28 bars over 4 weeks), expressed as a percentage. Participants with adherence ≥ 90% were included in the final (per-protocol) analysis.

### 2.4. Outcomes

The study evaluated two types of outcomes: a primary outcome (objective biochemical marker) and a secondary outcome (subjective psychometric assessment). All measurements were performed twice: at the screening stage (prior to the intervention, Visits 1–2) and within 24–48 h following completion of the 4-week intervention (Visit 6).

#### 2.4.1. Primary Outcome

The primary outcome was the change in salivary cortisol levels at 30–60 min after awakening (peak cortisol value) from baseline (Visit 1) to the final measurement (Visit 6). The selection of cortisol as the primary outcome was predicated on its status as the principal effector hormone of the HPA axis, reflecting the activity of the body’s stress system.

Saliva collection was performed by participants independently at home in the morning (between 6:00 and 10:00) at 30–60 min after awakening. One to three days prior to the scheduled collection, participants were provided with a sterile biomaterial container (Salivette^®^ Cortisol tube, Sarstedt, Germany) along with detailed written instructions, including the following requirements:

- refrain from eating, smoking, and toothbrushing for 60 min prior to collection;
- avoid physical exertion and emotional stress immediately before collection.

The collected saliva sample was labelled with the individual identification number, date, and time of collection, placed in a refrigerator (0… + 4 °C), and delivered to the laboratory no later than 24 h after collection.

Participants either delivered samples personally to the nearest medical office of the accredited laboratory LLC “CHROMOLAB” (Moscow, Russia) or handed them over to members of the research team, who transported the samples to the medical office located in close proximity to Plekhanov Russian University of Economics.

All participants collected morning saliva samples within the specified 30–60-min post-awakening window (between 6:00 and 10:00 a.m.) at both time points. No deviations from this window were recorded, and each participant adhered to the same post-awakening interval at baseline and at the final measurement (for example, a participant who collected the baseline sample 40 min after awakening also collected the final sample 40 min after awakening). Adherence to the collection protocol was verified through self-reported collection times recorded on the sample labels and confirmed during weekly in-person meetings.

Determination of salivary cortisol concentration was performed using high-performance liquid chromatography with tandem mass spectrometric detection (HPLC-MS/MS) on equipment at the laboratory of LLC “CHROMOLAB” (medical license No. Л041-01137-77/00383435 dated 6 August 2020). Analyses were conducted by laboratory staff who were unaware of sample group allocation. Results were expressed in ng/mL.

#### 2.4.2. Secondary Outcome

The secondary outcome was the change in perceived stress levels, assessed using the Perceived Stress Scale (PSS-14) (according to [64]). The selection of the PSS-14 was predicated on its widespread use in population-based and clinical studies for assessing the degree to which life situations are appraised by an individual as stressful [65]. The questionnaire measures the global level of subjective stress over the preceding month, which corresponds to the intervention time window (4 weeks).

Participants completed an electronic version of the PSS-14 questionnaire using the Yandex Forms tool (LLC “Yandex”, Moscow, Russia). A link to the questionnaire was sent to participants via individual messages on messaging platforms (Telegram or VK.com) no later than 1 day prior to the scheduled completion. Participants were instructed to complete the questionnaire independently, without consultation with others and without prior familiarization with the questions. Questionnaire completion took on average 5–7 min.

The PSS-14 questionnaire comprises 14 items assessing the frequency of stress-related experiences and thoughts over the past month. Responses are given on a 5-point Likert scale: 0—“never”, 1—“almost never”, 2—“sometimes”, 3—“fairly often”, 4—“very often”. Six items are reverse-coded. The total PSS-14 score was calculated as the sum of scores across all 14 items after reverse coding, ranging from 0 to 56 points. Interpretation of results was conducted in accordance with standardized threshold values: 0–13 points—low stress level, 14–26—moderate, 27–56—high stress level.

The Russian-language version of the PSS-14 has been validated by Zolotareva (2023) on a sample of 558 Russian-speaking respondents, demonstrating good internal consistency (Cronbach’s α = 0.841), a two-factor structure (distress and coping), and positive correlations with psychological distress as measured by the DASS-21 [66].

### 2.5. Procedures

The study comprised six sequential stages: remote screening, in-person screening with anthropometry and tube distribution, biomaterial collection and waiting for laboratory results, randomization and intervention commencement, 4-week intervention with weekly monitoring, and final testing. The schedule of visits and procedures is presented in Table 4.

### 2.6. Statistical Analysis

Statistical data analysis was performed using Jamovi software (version 2.6.44). Due to the detected deviation of all quantitative variables from normal distribution (tested using the Shapiro-Wilk test, visual inspection of Q-Q plots, and skewness and kurtosis coefficients), data are presented as median and interquartile range, Me [Q1; Q3].

The primary outcome analysis (change in salivary cortisol levels) was performed on a per-protocol basis (*n* = 28), including participants who completed the full 4-week protocol and provided all planned measurements at both time points (14 per group). The secondary outcome analysis (change in PSS-14 scores) was performed using an available-case approach (*n* = 29), including 15 participants in the intervention group and 14 in the control group who provided questionnaire data at both time points, regardless of whether the final saliva sample was available. A sensitivity analysis was conducted on the sample excluding the outlier participant (*n* = 27) to assess the robustness of the primary outcome findings. The sensitivity analysis was not pre-specified in the protocol and was conducted post hoc, after visual inspection of the distribution of change scores revealed one extreme value in the intervention group (Δ = −6.23 ng/mL).

For comparison of two independent groups, the Mann–Whitney U test was used; for evaluation of within-group dynamics, the Wilcoxon signed-rank test was applied. To assess intergroup effect size, the rank-biserial correlation coefficient (r) was used, with interpretation according to Cohen’s criteria: 0.1—small, 0.3—medium, 0.5—large effect. 95% confidence intervals for the rank-biserial coefficient were calculated using the bootstrap method with bias-corrected and accelerated adjustment based on 10,000 bootstrap replications. Correlation analysis was performed using Spearman’s rank correlation coefficient (ρ), with a 95% confidence interval calculated using Fisher’s z transformation. Differences were considered statistically significant at *p* < 0.05.

Given the observed baseline difference in age between the intervention and control groups, additional covariate-adjusted sensitivity analyses were conducted to assess the robustness of the primary between-group findings. For each outcome, post-intervention values were modelled as a function of treatment group, age, and the corresponding baseline value. Specifically, post-intervention cortisol was analyzed with treatment group, age, and baseline cortisol as predictors, whereas post-intervention PSS-14 was analyzed with treatment group, age, and baseline PSS-14 as predictors. These analyses were considered sensitivity analyses rather than primary analyses, given the small pilot sample and the non-normal distribution of the outcomes. Regression coefficients (β), 95% confidence intervals, and *p*-values were reported.

No multiplicity adjustment was applied across the primary, secondary, correlation, or sensitivity analyses. As this was a pilot study, the analyses were considered exploratory and were not intended to provide definitive confirmatory inference. Therefore, *p*-values were interpreted alongside effect sizes and their 95% confidence intervals, and results with *p*-values close to 0.05 were interpreted cautiously.

The planned sample size (*n* = 30 randomized participants) was determined by logistical and resource capabilities for recruitment and was not calculated for detection of a pre-specified minimal effect. The results obtained are considered preliminary and require confirmation in studies with larger sample sizes.

The sample size for a future confirmatory trial was estimated using the online sample-size calculator provided by the University of Vienna (https://homepage.univie.ac.at/robert.koch/samsize/, accessed on 13 September 2026). The probability of superiority, P(X > Y), was derived from the observed rank-biserial correlation coefficient using the formula

P(X > Y) = (1 + |r|)/2,(1)

A two-sided significance level of α = 0.05, a statistical power of 80%, and a 1:1 allocation ratio between the intervention and control groups were assumed. Sample sizes were calculated both without and with an allowance for a potential 15% attrition rate. The most conservative scenario was used to determine the proposed target number of participants for a future confirmatory trial. The calculation was intended solely to inform the design of a future adequately powered study and was not used as a retrospective assessment of the statistical power of the present pilot study.

The analytical approach was pre-specified in the study protocol. A per-protocol analysis was applied to the primary outcome (morning salivary cortisol), including only participants who completed the full 4-week protocol and provided all planned measurements at both time points (*n* = 28, 14 per group). An available-case analysis was applied to the secondary outcome (PSS-14), including all participants who provided questionnaire data at both time points, regardless of whether the final saliva sample was available (*n* = 29, 15 in the intervention group and 14 in the control group). Data imputation was not planned or performed, as the number of withdrawals was small (*n* = 2) and the reasons for withdrawal were unrelated to the intervention.

The choice of a per-protocol approach for the primary outcome was based on the following considerations. First, the study was designed as a pilot trial whose primary aim was to obtain an initial estimate of the effect of the intervention under conditions of actual exposure, rather than to estimate the effect of assignment to treatment under real-world conditions. For a nutritional intervention with a self-administered product, where adherence is a key determinant of biological exposure, the per-protocol estimate is more directly informative about the potential efficacy of the product under conditions of adequate intake—which was the primary question of interest in this pilot phase.

Second, the pilot nature of the study implies that the primary goal was signal detection—that is, to determine whether the intervention produces a measurable effect on the selected outcomes, and to obtain preliminary estimates of effect size and variability for planning a future confirmatory trial. For this purpose, a per-protocol analysis, which is not diluted by non-adherence or by participants who did not receive the intervention as intended, provides a more sensitive estimate of the potential effect. An intention-to-treat analysis, while methodologically preferable for confirmatory trials evaluating effectiveness, would have been less informative at this exploratory stage, particularly given the small sample size and the resulting limited statistical power.

## 3. Results

### 3.1. Participant Flow

Participant recruitment was conducted in May 2026. A total of 46 women responded to the remote screening, of whom 12 were excluded: 4—due to age, 2—due to BMI (1 below and 1 above the 17.0–29.9 kg/m^2^ range), 1—due to use of hormonal preparations, and 5—due to consumption of magnesium or vitamin C supplements. The remaining 34 participants provided a baseline saliva sample for determination of basal cortisol levels. Following receipt of laboratory results, a further 4 participants were excluded due to morning salivary cortisol levels below the threshold value of 1.5 ng/mL. Thus, 30 participants were randomized to the intervention and control groups in a 1:1 ratio and commenced the 4-week intervention.

During the course of the study, 2 participants withdrew: one from the control group—due to initiation of glucocorticoid therapy for medical indications (a post-hoc exclusion criterion); and one from the intervention group—due to inability to provide a final saliva sample in a timely manner because of an acute respiratory illness. The latter participant, however, did provide final PSS-14 questionnaire data. Accordingly, 28 participants (14 in each group) completed the full study protocol and were included in the analysis of the primary outcome (cortisol). For the secondary outcome (PSS-14), an available-case analysis included 29 participants (15 in the intervention group, 14 in the control group). A participant flow diagram is presented in Figure 1.

### 3.2. Intervention Adherence

In both groups, median adherence was 100%, with the interquartile range in the intervention group being [97.3; 100] and in the control group [96.4; 100]. All 28 participants included in the final analysis demonstrated adherence above the threshold value (≥90%), indicating that potential missed bar consumption did not bias the study results.

During the 4-week intervention, no adverse events related to bar consumption were reported. Participants did not report any gastrointestinal disturbances, allergic reactions, drowsiness, or other symptoms that could have been associated with the intake of the study products. All participants who completed the protocol (*n* = 28) reported good tolerability of the bars in both the intervention and control groups. Thus, the fortified date bar and the placebo bar demonstrated a favorable safety profile in the studied population of healthy young women.

### 3.3. Baseline Characteristics

Baseline characteristics of the participants are presented in Table 5. The analysis included 28 participants who completed the full protocol (per-protocol), with the exception of the PSS-14 measure, for which an available-case analysis was used (*n* = 29). Testing for normality of distribution of quantitative variables using the Shapiro–Wilk test, visual inspection of Q-Q plots, and skewness and kurtosis coefficients revealed deviation from normal distribution for all measures; accordingly, data are presented as median and interquartile range, Me [Q1; Q3], and the nonparametric Mann–Whitney U test was used for between-group comparisons.

The groups were comparable at baseline for most measures. No statistically significant between-group differences were found for BMI (*p* = 0.241), morning salivary cortisol (*p* = 0.982), or PSS-14 scores (*p* = 0.694). A difference in median age was observed between the intervention and control groups (20.5 vs. 23.5 years), which did not reach statistical significance (*p* = 0.102). However, given the small sample size (*n* = 14–15 per group), the study had low power to detect baseline imbalance, and a non-significant *p*-value should not be interpreted as evidence of true comparability. Age may relate to academic year and examination burden and could plausibly confound both stress perception and cortisol dynamics; this is addressed in the sensitivity analysis and discussed further.

All participants were full-time students at Moscow universities (undergraduate or graduate level), which ensured sample homogeneity with respect to educational level and professional status and allowed these variables to be excluded from the list of potential confounding factors.

### 3.4. Effects of the Intervention

#### 3.4.1. Effect on Salivary Cortisol Levels

To evaluate the effect of the intervention, an analysis of changes in morning salivary cortisol levels was conducted among the 28 participants who completed the full protocol (per-protocol). All participants (*n* = 28) adhered to the specified 30–60-min post-awakening collection window at both time points, with no deviations recorded; each participant also maintained the same post-awakening interval at baseline and post-intervention. The data are presented in Table 6.

Within-group analysis did not reveal a statistically significant change in cortisol levels in either group. In the intervention group, the median change was −0.64 [−1.64; 0.36] ng/mL (*p* = 0.173, Wilcoxon test), with the effect size corresponding to a medium level (*r* = 0.43). In the control group, the median change was 0.46 [−0.35; 1.02] ng/mL (*p* = 0.326, Wilcoxon test), with the effect size being small (*r* = −0.31). Between-group comparison of change magnitudes did not reveal a statistically significant difference between the groups (*p* = 0.104, Mann–Whitney U test). The effect size corresponded to a medium level (*r* = −0.37; 95% CI: −0.73; 0.11).

Visual analysis of the distribution of changes across groups revealed a wide scatter of individual responses to the intervention (Figure 2), including one extreme value (cortisol reduction of −6.23 ng/mL). After exclusion of this observation, the median change in cortisol in the intervention group was −0.62 [−1.14; 0.39] ng/mL. Between-group comparison of changes still did not reach statistical significance: *p* = 0.169 (Mann–Whitney U test), and was somewhat diminished compared with the original *p* = 0.104. The effect size remained at a medium level (*r* = −0.32; 95% CI: −0.70; 0.15). Thus, exclusion of the extreme value did not alter the main conclusions, allowing the influence of a single outlier on data interpretation to be ruled out.

#### 3.4.2. Effect on Perceived Stress Level

Analysis of the secondary outcome was performed on 29 participants who provided PSS-14 questionnaire data at both time points (available-case analysis). The analysis included 15 participants from the intervention group and 14 participants from the control group. The data are presented in Table 7.

Within-group analysis revealed a statistically significant reduction in PSS-14 scores in the intervention group: the median change was −4.0 points ([−8.0; −1.5]; *p* = 0.009, Wilcoxon test) with a large effect size (*r* = 0.80). In the control group, no statistically significant changes were observed: the median change was 0.0 [−4.0; 2.0] points (*p* = 0.624, Wilcoxon test) with a small effect size (*r* = 0.18). Between-group comparison of change magnitudes did not reach statistical significance: the median change in the intervention group was −4.0 [−8.0; −1.5] points versus 0.0 [−4.0; 2.0] points in the control group (*p* = 0.073, Mann–Whitney U test). The effect size corresponded to a medium level (*r* = −0.40; 95% CI: −0.75; 0.07).

Visual analysis of the distribution of final PSS-14 scores (Figure 3) revealed one participant in the intervention group with a high value (37 points); however, between-group differences in both baseline and final levels remained statistically non-significant (*p* = 0.843 and *p* = 0.061, respectively). At the same time, the distribution of changes was homogeneous and contained no outliers, allowing the between-group comparison of changes (*p* = 0.073) to be interpreted as robust.

#### 3.4.3. Covariate-Adjusted Sensitivity Analysis

To examine whether the observed baseline age imbalance influenced the findings, covariate-adjusted sensitivity analyses were performed. For morning salivary cortisol, adjustment for age and baseline cortisol resulted in a between-group coefficient of β = −0.89 (95% CI: −2.11; 0.34, *p* = 0.148), indicating that the direction of the between-group effect remained unchanged, although the adjusted difference was not statistically significant. Age was not significantly associated with post-intervention cortisol (β = −0.07, 95% CI: −0.32; 0.19, *p* = 0.590), whereas baseline cortisol was a strong predictor of post-intervention cortisol (β = 0.93, 95% CI: 0.52; 1.33, *p* < 0.001).

For PSS-14, adjustment for age and baseline PSS-14 similarly preserved the direction of the between-group effect. The adjusted group coefficient was β = −3.17 (95% CI: −8.02; 1.69, *p* = 0.190). Age showed a trend toward an inverse association with post-intervention PSS-14 scores (β = −0.88, 95% CI: −1.87; 0.10, *p* = 0.077), while baseline PSS-14 was significantly associated with post-intervention scores (β = 0.52, 95% CI: 0.16; 0.88, *p* = 0.007). Overall, adjustment for age did not reverse the direction of the observed between-group effects, although the small sample size resulted in wide confidence intervals and limited precision.

### 3.5. Correlation Analysis

To assess the relationship between objective and subjective changes in stress measures, Spearman’s correlation analysis was performed between the magnitude of change in salivary cortisol levels (Δ cortisol) and the magnitude of change in Perceived Stress Scale PSS-14 scores (Δ PSS-14) across the entire sample (*n* = 28).

No statistically significant correlation was found between the measures (ρ = −0.134; 95% CI: −0.49; 0.25; *p* = 0.496). This result indicates the absence of a linear relationship between the dynamics of the objective biomarker (cortisol) and subjective stress assessment (PSS-14) in the studied sample.

Similarly, no statistically significant correlation was found between baseline cortisol and PSS-14 levels across the entire sample (ρ = −0.148; 95% CI: −0.49; 0.25; *p* = 0.452). This finding confirms the independence of biochemical and psychological stress indicators and justifies the appropriateness of their separate analysis as primary and secondary outcomes.

### 3.6. Sample Size Calculation for a Future Confirmatory Trial

Based on the between-group rank-biserial correlation observed for change in morning salivary cortisol in present pilot study (*r* = −0.37), the corresponding probability of superiority was P(X > Y) = 0.685. With a two-sided α = 0.05 and 80% power, the online University of Vienna sample-size calculator indicated that 39 evaluable participants per group (78 in total) would be required for a future two-arm trial with equal allocation. Allowing for 15% attrition increased the required recruitment target to 44 participants per group (88 in total). To account for the uncertainty associated with the effect estimate from the small pilot sample, more conservative scenarios were also examined. Assuming P(X > Y) = 0.660 (if *r* = −0.32, as shown by the sensitivity analysis), 52 evaluable participants per group (104 in total) would be required, increasing to 58 participants per group (116 in total) after allowing for 15% attrition. Under the most conservative scenario considered, P(X > Y) = 0.650, 59 evaluable participants per group (118 in total) would be required, corresponding to a recruitment target of 66 participants per group (132 in total) after accounting for 15% attrition. Thus, based on the conservative planning scenario, a future confirmatory trial should aim to recruit approximately 132 participants (66 per group) to provide 80% power.

## 4. Discussion

The present pilot RCT was aimed at evaluating the effect of 4-week consumption of a date bar fortified with magnesium, vitamin C, inulin, and grape seed extract on the level of subjectively perceived stress and morning salivary cortisol in healthy female university students.

Beginning the discussion, it is important first to highlight the high level of adherence to the study protocol among participants: median adherence in both groups was 100%, and all participants included in the final per-protocol analysis demonstrated a rate of ≥90%. This finding indicates that the selected carrier product (the date bar) proved convenient for integration into the daily lives of young women. The following factors may have contributed to participant adherence to the intervention: the pleasant organoleptic properties of the bar and the absence of any need for additional preparation prior to consumption; the active use of digital tools for reminders (messaging platform notifications and brief surveys to confirm bar intake) in accordance with the communication habits of the target age group [67]; and weekly in-person meetings for bar distribution. The main reasons for missed doses recorded during the study were not systematic but rather situational in nature, predominantly related to temporary travel outside the city, during which the bars were left at home. This suggests that organizational logistical barriers, rather than biological or behavioral factors (such as product rejection or side effects), were the main barrier to perfect adherence under the conditions of a pilot project.

The results of the primary outcome of the intervention proved to be ambiguous. Although the intervention group showed a reduction in morning cortisol levels (median change of −0.64 ng/mL), within-group analysis demonstrated that this reduction did not reach statistical significance (*p* = 0.173, Wilcoxon test), with a medium within-group effect size (*r* = 0.43). In the control group, cortisol, conversely, demonstrated a slight increase (median change of +0.46 ng/mL), with the within-group effect size being small (*r* = −0.31) and the changes statistically non-significant (*p* = 0.326).

Between-group comparison of change magnitudes, although not reaching the threshold of statistical significance (*p* = 0.104, Mann–Whitney U test), demonstrated a moderate effect size (*r* = −0.37; 95% CI: −0.73; 0.11), suggesting a possible difference in dynamics between the groups that was likely not detected as statistically significant due to limited sample power. The sensitivity analysis performed with the exclusion of one extreme value in the intervention group (a reduction of −6.23 ng/mL in a participant with a baseline elevated cortisol level of 12.6 ng/mL) showed that the between-group difference remained statistically non-significant (*p* = 0.169), with a medium effect size (*r* = −0.32; 95% CI: −0.70; 0.15). Thus, the exclusion of the outlier did not alter the main conclusion: the between-group comparison remains non-significant and the confidence interval remains wide and includes zero.

The absence of a significant effect on cortisol may be interpreted from several perspectives. Firstly, the pilot nature of the study and the associated limited sample size (*n* = 28 for the per-protocol analysis) must be taken into account. As demonstrated, the observed tendency towards cortisol reduction in the intervention group compared with the control group corresponds to a medium effect size, which with a larger sample might have reached statistical significance. That is, the study was likely underpowered to detect moderate changes in basal cortisol, and the present data cannot determine whether such changes are present.

Secondly, the intervention duration (4 weeks) may have been insufficient for changes in nutritional status (particularly magnesium and vitamin C) to lead to restructuring at the level of the HPA axis. Studies demonstrating the effects of magnesium and vitamin C on cortisol metabolism often involve longer observation periods (from 8 to 24 weeks) [57,68]. Furthermore, in our study, participants were healthy young women with normal basal cortisol levels (median 3.89 and 3.77 ng/mL in the groups), whereas in other studies, subjects are most often in a state of acute or chronic stress [68,69,70].

Thirdly, the choice of saliva collection time (peak value at 30–60 min after awakening) reflects HPA-axis reactivity to awakening and is simultaneously a highly sensitive and highly variable measure, dependent on numerous situational factors: time of awakening, quality of preceding sleep, physical activity, and so forth [71]. It is possible that for assessing the long-term anti-stress effect of nutrients, the diurnal cortisol profile with multiple time points is more informative [72].

Finally, it cannot be ruled out that the selected dosages of magnesium (240 mg of elemental magnesium per day) and vitamin C (300 mg) were insufficient to achieve a physiologically significant effect on the HPA axis in healthy individuals. In studies where a positive effect of magnesium on stress reactivity was observed, daily doses reached 500 mg of elemental magnesium [70], while the effects of vitamin C on cortisol are registered at dosages of approximately 500–1000 mg [16,73,74].

Thus, the absence of a statistically significant between-group effect on cortisol should be interpreted in light of the limited statistical power of the pilot sample and the wide confidence interval of the effect estimate, rather than as evidence of the absence of an effect. The present data do not allow conclusions to be drawn about the efficacy or inefficacy of the intervention with respect to cortisol, and longer-term studies with larger sample sizes are required. More sensitive and informative biomarkers should also be selected, including additional parameters (for example, the ratio of cortisol and dehydroepiandrosterone [75]).

Analysis of the secondary outcome—the level of subjectively perceived stress as measured by the PSS-14 scale—revealed a statistically significant reduction in the intervention group: within-group analysis demonstrated a decrease in the median PSS-14 score by −4.0 points (*p* = 0.009, Wilcoxon test) with a large effect size (*r* = 0.80). This magnitude of change is consistent with the range reported in the literature for the minimal clinically important difference on the PSS [76], suggesting a potentially meaningful improvement in subjective stress perception among participants consuming the fortified bar. In the control group, the median change was 0.0 points (*p* = 0.624), with a small effect size (*r* = 0.18), indicating the absence of dynamics. Between-group comparison of change magnitudes, although not reaching the threshold of statistical significance (*p* = 0.073, Mann–Whitney U test), demonstrated a medium effect size (*r* = −0.40; 95% CI: −0.75; 0.07). This pattern is consistent with a directionally consistent but statistically non-significant trend, which may reflect limited statistical power and should be interpreted with caution given the exploratory nature of this study.

Our findings are consistent with data from systematic reviews and RCTs confirming the positive effects of magnesium on psychological measures of stress, anxiety symptoms, and depression. A systematic review [56] demonstrated the beneficial effect of magnesium supplementation on mild anxiety in vulnerable individuals; an RCT [77] showed a reduction in moderate stress levels in patients with fibromyalgia; an RCT [78] confirmed the efficacy of magnesium supplementation in reducing symptoms of mild-to-moderate depression. Vitamin C has also been documented in the literature as a nutrient that has demonstrated efficacy in reducing anxiety, stress, and improving mood in such social groups as students [79,80], patients with type 2 diabetes mellitus [54], and working adults [81]; vitamin C supplementation has also been recognized as an effective adjunctive therapy for patients suffering from major depressive disorder [82]. Thus, the reduction in subjective stress observed in our study among participants in the intervention group is consistent with the accumulated body of evidence in the field of nutritional psychophysiology, thereby confirming the biological plausibility of the observed effects. At the same time, the complex composition of the date bar, which in addition to magnesium and vitamin C also includes inulin and grape seed extract, suggests the possibility of a synergistic interaction between components, which may potentiate the anti-stress potential and warrants further investigation in specialized studies.

It should be noted that in the covariate-adjusted sensitivity analysis, age showed a trend toward an inverse association with post-intervention PSS-14 scores (β = −0.88, 95% CI: −1.87; 0.10, *p* = 0.077), suggesting that older participants tended to report lower perceived stress after the intervention, independently of group allocation. However, this association did not reach statistical significance, and the confidence interval included zero. Importantly, because the analysis did not include an age-by-group interaction term, it cannot be determined whether the intervention effect itself differed by age; the observed pattern reflects a main effect of age on the outcome rather than a differential response to the intervention.

Several contextual factors may help explain this pattern, although they remain speculative. The main part of the intervention was conducted in June, a period when Russian university students take examinations, and final-year students defend their theses. It is therefore possible that older participants—who were more likely to be in their final year—experienced a natural decline in stress following the completion of thesis preparation, which had been ongoing throughout the spring. In contrast, younger students may have still faced ongoing academic demands in July, such as summer practical training, meaning that for them the academic year had not yet ended and the associated relief had not yet occurred. These contextual differences could have contributed to the observed association between age and post-intervention PSS-14 scores. However, these explanations are post hoc and cannot be tested with the present data; they are offered as possible interpretations rather than as established mechanisms. The small sample size (*n* = 29 for PSS-14) further constrains the precision of the estimate, and the finding should therefore be regarded as hypothesis-generating rather than confirmatory.

Of particular note is the discrepancy observed in the present study between the dynamics of the objective (cortisol) and subjective (PSS-14) stress measures. Correlation analysis revealed no significant relationship between changes in cortisol levels and changes in PSS-14 scores (ρ = −0.134; 95% CI: −0.49; 0.25; *p* = 0.496); nor was any association found between baseline cortisol levels and baseline PSS-14 scores (ρ = −0.148; 95% CI: −0.49; 0.25; *p* = 0.452), indicating the relative independence of these two measures. This finding is consistent with numerous studies demonstrating that subjective stress perception and objective neuroendocrine markers often correlate weakly or moderately [83,84].

The discrepancy between the level of perceived stress and cortisol levels may be due to the fact that cortisol levels reflect the activity of the HPA axis in a narrow time window (morning peak), whereas the PSS-14 scale assesses the perception of stress over the previous month, which makes these measures complementary, but not interchangeable.

It is also likely that the mechanisms through which nutrients influence subjective well-being involve not only the HPA axis but also other pathways that are not reflected in morning cortisol levels. These include modulation of neurotransmitter systems [85], improvement in sleep quality [29], reduction in fatigue [86], as well as anti-inflammatory effects that may influence mood and general well-being [87,88]. In addition, both inulin and grape seed extract may act through pathways that are not captured by morning cortisol measurement. Inulin, as a prebiotic fiber, can modulate the composition and metabolic activity of the gut microbiota, increasing the production of short-chain fatty acids, which possess neuroactive properties and are involved in gut–brain axis signaling [89]. Grape seed polyphenols, in turn, may influence the gut microbiota composition and exert systemic anti-inflammatory and antioxidant effects, including modulation of cytokine production and reduction of oxidative stress [61,62]. These microbiome-mediated and anti-inflammatory mechanisms may contribute to the improvement in subjective stress perception observed in the intervention group, even in the absence of a detectable change in morning cortisol. It should be emphasized that because the fortified bar contained four bioactive ingredients, this study cannot determine which component—or which combination of components—was responsible for the observed changes; therefore, the observed effects should be attributed to the composition as a whole.

Strengths of the present work include: a rigorous double-blind placebo-controlled design with stratified randomization based on basal cortisol levels; high participant adherence to the protocol; the use of both objective (biochemical) and subjective (psychometric) methods of stress assessment, which allowed for a multidimensional picture of the effect; and the selection of a carrier product with high scalability potential due to its organoleptic acceptability, accessibility, and ease of integration into the daily diet. The data obtained contribute to the development of the evidence base in the field of nutritional stress prevention and may serve as a foundation for planning full-scale RCTs with larger sample sizes and longer observation periods. The observed rank-biserial correlation corresponded to a probability of superiority of 0.685, which would require 39 evaluable participants per group to achieve 80% power at a two-sided α = 0.05. However, given the small sample size of the pilot study and the considerable uncertainty regarding the effect estimate, direct use of the observed effect may lead to an overly optimistic sample size estimate. More conservative scenarios were therefore considered. Assuming a probability of superiority of 0.660, 52 evaluable participants per group would be required, whereas a probability of superiority of 0.650 would require 59 evaluable participants per group. After accounting for 15% attrition, the target sample size in the latter scenario would be 132 participants (66 per group). Thus, approximately 132 participants represent a reasonable conservative recruitment target for a future confirmatory trial examining the effect of the intervention on morning salivary cortisol levels.

Alongside the aforementioned strengths, several limitations must be taken into account when interpreting the results of the present study. First and foremost, the pilot nature of the work resulted a limited sample size (*n* = 28 for the per-protocol analysis), which substantially restricts statistical power and precludes the detection of moderate effects—a consideration particularly relevant to cortisol. The results should be regarded as preliminary, requiring confirmation in larger samples. Secondly, the relatively short intervention duration (4 weeks) may have been insufficient for the manifestation of physiologically significant changes at the level of the HPA axis, particularly in healthy individuals with normal basal cortisol levels. Future studies with longer observation periods (8–12 weeks) would allow for assessment of the long-term efficacy of the intervention and the sustainability of the effect. Thirdly, dietary intake of magnesium, vitamin C, polyphenols, and other nutrients was not monitored during the study. Although participants were advised not to alter their habitual diet or physical activity levels, no dietary records, food frequency questionnaires, or physical activity questionnaire were collected at baseline or at the end of the intervention. It therefore cannot be excluded that between-group differences in physical activity and habitual intake of nutrients—or changes in intake over the 4-week period—contributed to the observed dynamics, particularly for the subjective stress outcome. It should also be taken into account that, in the present study, the intervention was evaluated in comparison with a placebo bar rather than with the complete absence of a bar. Such a design makes it possible to isolate the effect of fortification from the effect of the food matrix itself; however, it does not allow assessment of whether the date bar as such makes an independent contribution to the dynamics of the studied stress indicators. Fourthly, the selection of morning cortisol as the sole objective biomarker of stress substantially narrowed the picture of the nutritional intervention’s impact on the stress system. Future studies should explore additional biomarkers (evening cortisol, diurnal profile, cortisol-to-dehydroepiandrosterone ratio). In addition, it is well documented that awakening cortisol varies substantially from day to day within the same individual. Although all participants adhered to the specified 30–60-min post-awakening window at both time points, with no deviations recorded, and maintained the same post-awakening interval at baseline and post-intervention, this does not eliminate day-to-day biological variability. Future studies should consider repeated sampling across consecutive days to improve the precision of effect estimates. Fifthly, the lack of laboratory confirmation of serum magnesium and vitamin C levels at baseline and upon intervention completion precludes assessment of the extent to which the bars effectively replenished any potential deficiency of these micronutrients among participants. Sixth, this study did not conduct a formal assessment of blinding integrity. Although allocation concealment, independent randomization, and blinded laboratory analysis were rigorously implemented, participants and personnel were not asked to guess their group assignment at the end of the study. Because magnesium citrate and ascorbic acid can impart a perceptible taste, there is an unassessed risk of partial unblinding, which could have influenced subjective stress reports. Future studies should include an end-of-study blinding-assessment item to quantify the success of blinding. Finally, the use of per-protocol and available-case analyses rather than intention-to-treat should also be acknowledged as a limitation. Although the analytical approach was pre-specified in the protocol and the number of withdrawals was small (*n* = 2), with reasons unrelated to the intervention, per-protocol analyses may introduce attrition bias and may overestimate the intervention effect, particularly if adherence or withdrawal is related to unmeasured prognostic factors. A future confirmatory trial should be designed with an intention-to-treat analysis as the primary analytical approach, with per-protocol analysis reported as a secondary, sensitivity analysis.

Several limitations related to the study population and recruitment should also be acknowledged. Participants were recruited through social media and messaging platforms, which resulted in a convenience sample rather than a probabilistically selected or clinically referred one. Such samples are prone to self-selection bias: women who respond to study advertisements may differ systematically from the broader population of female university students in terms of health consciousness, motivation to participate in nutritional interventions, or baseline levels of stress and health-related behaviors. Furthermore, the study population was restricted to healthy female university students aged 18–28 years residing in Moscow or the Moscow Region, with no diagnosed mental disorders or chronic endocrine diseases. While these criteria ensured sample homogeneity and reduced confounding, they also substantially narrow the generalizability of the findings. The results may not be directly extrapolated to male students, to women of other age groups, to individuals with diagnosed psychiatric or endocrine conditions, or to populations outside the Moscow region with different dietary patterns, stress levels, and access to healthcare. In addition, the exclusion of participants with morning salivary cortisol below 1.5 ng/mL further narrowed the population to women with preserved HPA axis activity. Taken together, these factors mean that the present findings should be regarded as applicable primarily to the specific population studied, and confirmation in more diverse and representative samples is required before any broader conclusions can be drawn.

## 5. Conclusions

The effect of 4-week consumption of a date bar fortified with magnesium (240 mg), vitamin C (300 mg), inulin (1000 mg), and grape seed extract (100 mg) on stress parameters in healthy female university students proved to be ambiguous and dependent on the chosen outcome measure. With respect to the objective biomarker—morning salivary cortisol—no statistically significant changes were revealed in either within-group (*p* = 0.173) or between-group (*p* = 0.104) comparisons; however, the intervention group demonstrated a tendency towards reduction in this parameter (median difference of −0.64 ng/mL) with a medium effect size for both within-group (*r* = 0.43) and between-group (*r* = −0.37) comparisons. This pattern is consistent with a possible but statistically non-significant trend that may reflect limited statistical power. With respect to subjectively perceived stress, in contrast, within-group analysis demonstrated a statistically significant reduction in PSS-14 scores in the intervention group (*p* = 0.009) with a large effect size (*r* = 0.80). However, the between-group comparison of change scores did not reach the conventional significance threshold (*p* = 0.073), although the effect size was moderate (*r* = −0.40). Taken together, these findings are consistent with a possible beneficial effect of the intervention on perceived stress, but they should be interpreted with caution given the exploratory nature of the study and the limited statistical power to detect between-group differences.

Correlation analysis revealed no significant relationship between changes in cortisol levels and changes in PSS-14 scores (ρ = −0.134; *p* = 0.496), nor between baseline levels of these measures (ρ = −0.148; *p* = 0.452), confirming the relative independence of objective neuroendocrine and subjective psychometric stress markers and justifying the need for their separate evaluation in nutritional support studies.

Thus, the data obtained suggest a possible effect of the fortified date bar on subjective stress perception, alongside a promising yet statistically non-significant trend with respect to the objective biomarker. These findings warrant further investigation in studies with an optimized design, including larger samples, longer intervention periods, and repeated cortisol sampling. Taken together, the results support the view that a fortified date bar may warrant further investigation as a potentially feasible, acceptable, and economically accessible component of primary stress prevention strategies in young health women populations, although this possibility remains to be tested in adequately powered confirmatory trials before any broader conclusions can be drawn.

## Figures and Tables

**Figure 1:**
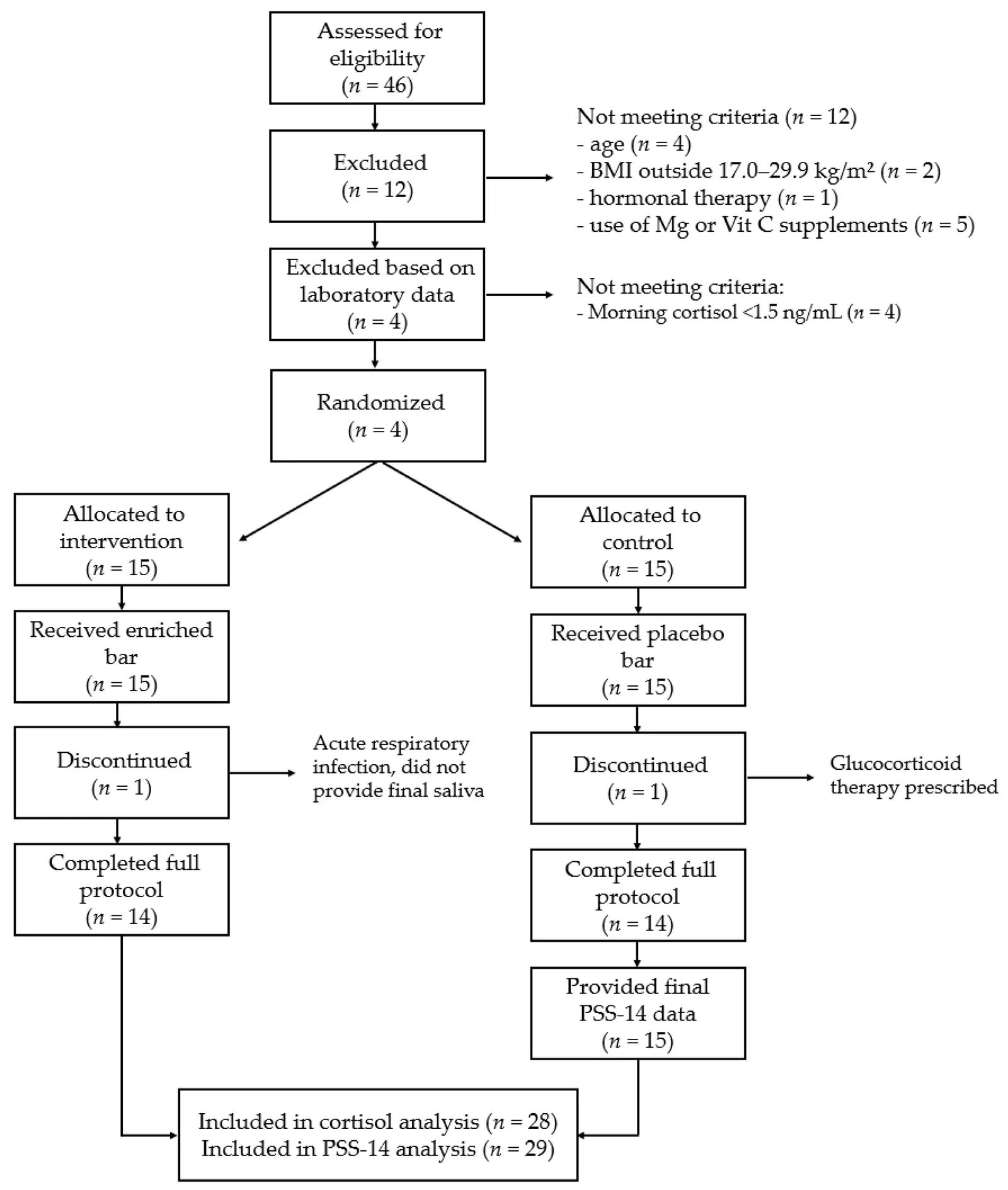
CONSORT flow diagram of the study.

**Figure 2:**
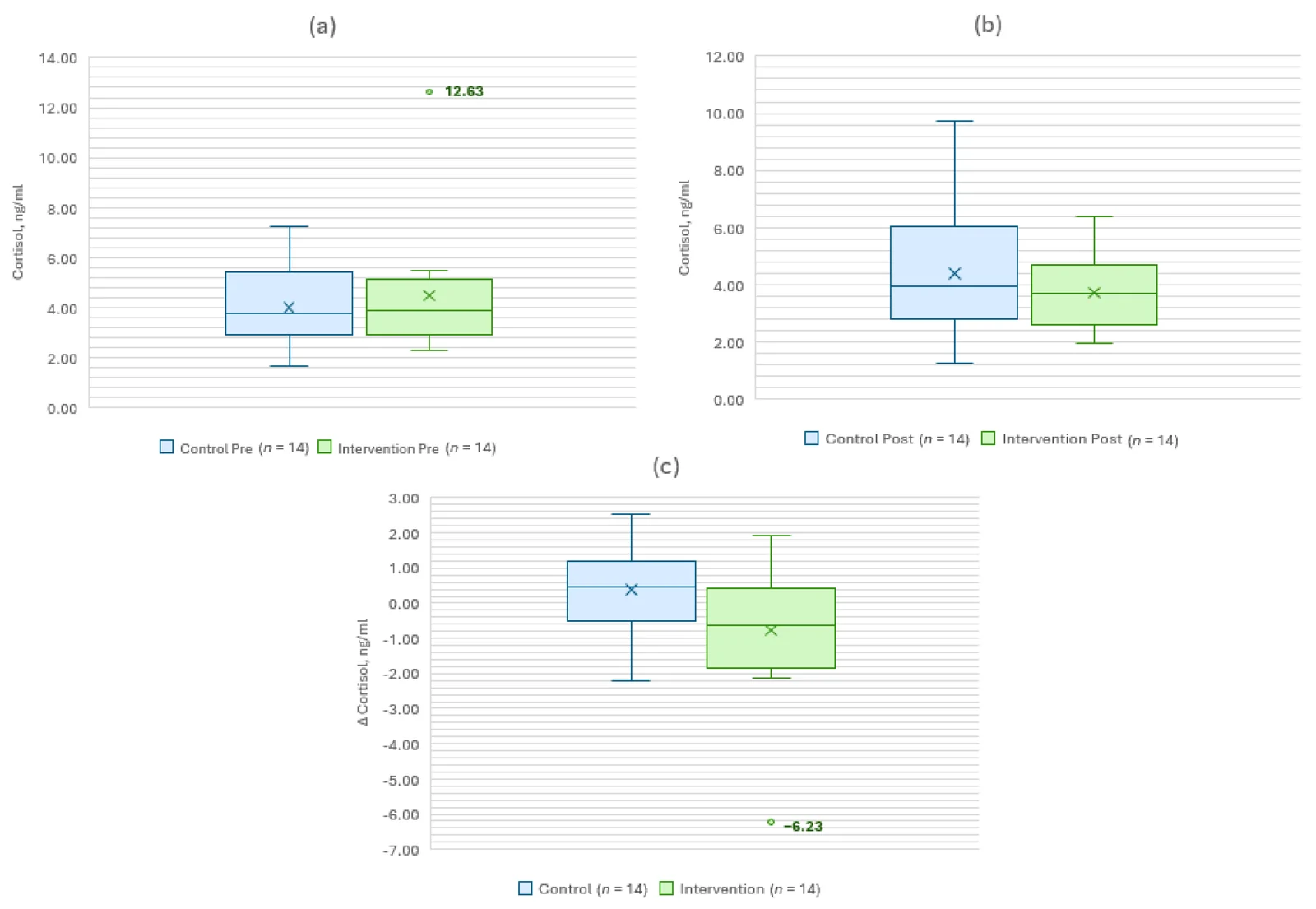
Changes in salivary cortisol levels before and after the 4-week intervention in the intervention and control groups. (**a**) Baseline cortisol levels. One participant in the intervention group had a markedly elevated baseline value (12.6 ng/mL). No statistically significant between-group differences were observed (*p* = 0.982, Mann–Whitney U test). (**b**) Post-intervention cortisol levels. (**c**) Changes in cortisol levels. In all panels, the horizontal line within each box denotes the median, the box represents the interquartile range, the whiskers extend to the most extreme values within 1.5× the interquartile range, and the «×» marker denotes the mean, the green circle indicates an outlier. One extreme outlier was identified in the intervention group (Δ = −6.23 ng/mL). Sensitivity analysis excluding this outlier did not change the main conclusions.

**Figure 3:**
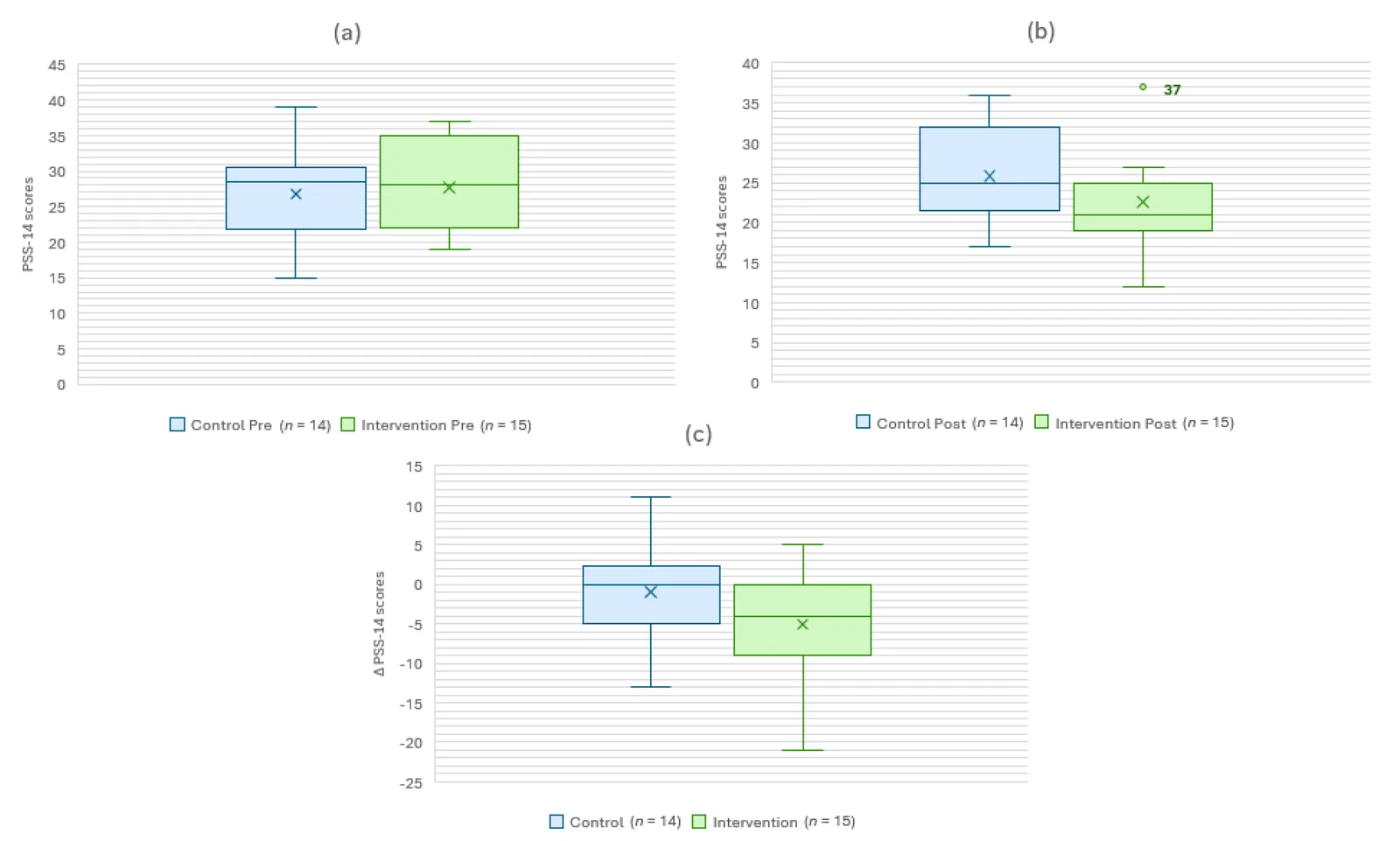
Changes in perceived stress scale (PSS-14) scores before and after the 4-week intervention in the intervention and control groups. (**a**) Baseline stress levels. (**b**) Post-intervention stress levels. One participant in the intervention group had a high post-intervention score (37 points); however, no statistically significant between-group differences were observed (*p* = 0.137). (**c**) Changes in PSS-14 scores. In all panels, the horizontal line within each box denotes the median, the box represents the interquartile range, the whiskers extend to the most extreme values within 1.5× the interquartile range, and the «×» marker denotes the mean, the green circle indicates an outlier. The distribution of changes contained no extreme outliers, indicating a relatively homogeneous response to the intervention in both groups.

**Table 1:** Inclusion and Exclusion Criteria.

Inclusion Criteria	Justification	Exclusion Criteria	Justification
Female	High prevalence of stress-related disorders among women; control of gender differences in HPA axis activity	Diagnosed psychiatric disorders	Possible association with altered HPA axis activity
Age 18–28 years	Minimizing age-related changes in endocrine regulation and ensuring sample homogeneity	Use of glucocorticoids or hormonal medications	Confounder
BMI 17.0–29.9 kg/m^2^	Elimination of the influence of extreme BMI values on basal cortisol levels and stress reactivity	Use of dietary supplements containing magnesium, vitamin C, or polyphenols	Avoiding the cumulative effect
Living in Moscow region	Logistics accessibility	Chronic endocrine disorders	Accompanied by disturbances in cortisol regulation
Provided informed consent	Mandatory ethical requirement	Baseline salivary cortisol < 1.5 ng/mL	Exclusion of extremely low values indicating HPA axis suppression
-	-	Allergy to bar components	Ensuring the safety of participants

**Table 2:** Composition of the bars.

Ingredient	Functional Purpose	Fortified Bar, %	Placebo Bar, %
Date paste	Product base, source of natural sugars	50.0	50.0
Raisins	Sweetener, texture enhancer	15.0	15.0
Cocoa	Flavoring component	10.0	10.0
Sorbitol syrup	Plasticizer, humectant	5.0	5.0
Almonds	Texturizer, source of fat	5.0	5.0
Magnesium citrate	Active ingredient (Mg^2+^, 240 mg/serving)	6.0	-
L-ascorbic acid	Active ingredient (vitamin C, 300 mg/serving)	1.2	-
Grape seed extract (powder)	Active ingredient (polyphenols, 100 mg/serving)	0.4	-
Inulin	Prebiotic, dietary fiber	4.0	-
Maltodextrin	Filler, volume compensation	3.4	15.0

**Table 3:** Nutritional value of the bars.

Parameter	Fortified Bar	Placebo Bar
Energy, kcal	70.0	79.0
Energy, kJ	293.0	331.0
Protein, g	1.2	1.2
Fat, g	0.95	0.95
Carbohydrates, total, g	14.4	17.1
sugars, g	10.0	10.0
polyols (sorbitol), g	0.88	0.88
Dietary fiber, g	3.1	2.2

**Table 4:** Schedule of visits and study procedures.

Stage/Visit	Time	Participant’s Actions	Researcher’s Actions
Remote Screening	Week-2	Completing an online questionnaire	Checking compliance with preliminary criteria
In-person screening (Visit 1)	Week-1	Signing of informed consent; provision of anthropometric data (height, weight); receipt of a saliva container and verbal/written instructions for collection	Height and weight measurement, BMI calculation; inclusion/exclusion criteria verification; provision of a Salivette^®^ Cortisol tube and detailed saliva collection instructions
Biospecimen Collection	On a day convenient for the participant during Week-1	Self-collection of morning saliva (30–60 min after waking up, between 6:00 and 10:00 a.m.) and delivery of the sample to the CHROMOLAB LLC laboratory (independently or through the researcher)	Monitoring compliance with sample collection conditions; collecting samples from participants and transporting them to the laboratory under temperature control
Waiting for lab results and randomization	3–5 days after saliva collection	Participants do not perform any actions and await an invitation for the next visit	Receipt of cortisol results from the laboratory; stratified randomization based on basal cortisol levels; formation of individual packages with bars for each participant in accordance with the randomization result; preparation for distribution
Baseline testing and intervention start (Visit 2)	Week 0 (intervention start day, 1–3 days after randomization)	Completion of the baseline PSS-14 questionnaire (electronic form Yandex Forms); receipt of an individual package with bars for one week (7 bars)	PSS-14 completion check; distribution of bars; verbal instructions on the intake regimen; answers to participants’ questions
Weekly Meetings (Visits 3–5)	Weeks 1, 2, and 3	Daily bar consumption with a brief online report via a messenger survey	Adherence monitoring; distribution of bars for the next week (7 units); consumption reminder
Final testing (Visit 6)	Week 4 (24–48 h after the last bar)	Repeat morning saliva collection (same conditions); repeat PSS-14 completion	Obtaining final saliva samples; checking PSS-14 completion; post-study debriefing (informing the participant which group she was assigned to)

**Table 5:** Baseline characteristics of the participants.

Parameter	Main Group	Control Group	*p*-Value ^1^
Age, years (*n*)	20.5 [20.0; 21.0] (14)	23.5 [20.3; 25] (14)	0.102
BMI, kg/m^2^ (*n*)	19.9 [19.2; 21.4] (14)	21.8 [19.9; 23.4] (14)	0.241
Salivary cortisol (morning), ng/mL, (*n*)	3.89 [3.0; 5.04] (14)	3.77 [3.03; 5.14] (14)	0.982
PSS-14, scores (*n*)	28.0 [23.5; 33.5] (15)	28.5 [22.0; 30.0] (14)	0.694

^1^ Calculated using the Mann-Whitney U test.

**Table 6:** Dynamics of saliva cortisol levels.

Parameter	Main Group (*n* = 14)	Control Group (*n* = 14)	*p*-Value (Between Groups) ^1^	Effect Size *r*
Baseline level, ng/mL	3.89 [3.0; 5.04]	3.77 [3.03; 5.14]	0.982	0.01
Outcome level, ng/mL	3.68 [2.82; 4.54]	3.97 [2.92; 5.56]	0.597	−0.12
Δ, ng/mL	−0.64 [−1.64; 0.36]	0.46 [−0.35; 1.02]	0.104	−0.37 95% CI ^3^: −0.73; 0.11
*p*-value (within-groups) ^2^	*p* = 0.173 (*r* = 0.43)	*p* = 0.326 (*r* = −0.31)	-	-

^1^ Calculated using the Mann–Whitney U test. ^2^ Calculated using the Wilcoxon test. ^3^ Calculated using the bootstrap method with bias-corrected and accelerated adjustment based on 10,000 bootstrap replications.

**Table 7:** Dynamics of the level of perceived stress (PSS-14).

Parameter	Main Group (*n* = 15)	Control Group (*n* = 14)	*p*-Value (Between Groups) ^1^	Effect Size *r*
Baseline level, scores	28.0 [23.5; 33.5]	28.5 [22.0; 30.0]	0.694	0.09
Outcome level, scores	21.0 [19.5; 24.5]	25.0 [22.3; 30.5]	0.137	−0.32
Δ, scores	−4.0 [−8.0; −1.5]	0.0 [−4.0; 2.0]	0.073	−0.40 95% CI ^3^: −0.75; 0.07
*p*-value (within-groups) ^2^	*p* = 0.009 (*r* = 0.80)	*p* = 0.624 (*r* = 0.18)	-	-

^1^ Calculated using the Mann-Whitney U test. ^2^ Calculated using the Wilcoxon test. ^3^ Calculated using the bootstrap method with bias-corrected and accelerated adjustment based on 10,000 bootstrap replications.

## Data Availability

The original contributions presented in this study are included in the article and Supplementary Materials. Further inquiries can be directed to the corresponding authors.

## Supplementary Materials

The following supporting information can be downloaded at https://www.mdpi.com/article/10.3390/nu18193134/s1, CONSORT Checklist.

## Abbreviations

The following abbreviations are used in this manuscript:

BMI	Body Mass Index
HPA	Hypothalamic–Pituitary–Adrenal (axis)
HPLC-MS/MS	High-Performance Liquid Chromatography-Tandem Mass Spectrometry
NMDA	N-methyl-D-aspartate (receptor)
PSS-14	Perceived Stress Scale, 14-item
RCT	Randomized Controlled Trial
